# Hairy Cell Leukemia and Bladder Cancer in a Patient: Relation with Dye Exposure and Review of the Literaure

**DOI:** 10.1155/2009/812960

**Published:** 2009-04-29

**Authors:** Semra Paydas

**Affiliations:** Department of Oncology, Faculty of Medicine, Cukurova University, Adana 01330, Turkey

## Abstract

Chemical exposure is important in the etiology of some cancers. Dye or solvent exposures are important both in bladder cancer (BC) and hairy cell leukemia (HCL). Here a case with BC and HCL has been presented and literature has been reviewed.

## 1. Case Report

A 53-year-old man referred to our unit due to severe pancytopenia and splenomegaly. He had the history of pain during urination and hematuria for the last 2 years. He admitted to a urologist and bladder malignancy had been thought with abdominopelvic ultrasonographic examination. This US showed bladder wall thickness at the right-lower portion and massive splenomegaly with space occupying lesions in it, and it had been reported as BC and massive splenomegaly, probably spleen metastasis due to BC. PET/CT had been done and it showed thickening in bladder wall and splenomegaly with homogenous, low FDG uptake.

TUR had been performed due to BC by his urologist. After surgery, Mitomycin C instillation was given weekly for 4 weeks and one injection in every six months for four times is then given. After these he admitted to our unit with pancytopenia and splenomegaly.

His personal history was interesting for chemical exposure: he was interested with music and he was guitar player for years. He had the history of sellulosic dye exposure due to his professional job. He had the history of smoking and alcohol ingestion daily (whisky and Turkish raki) for 25 years.

Physical examination showed pallor and splenomegaly 8 cm below the left costal margin.

Abnormal laboratory tests are Hb: 7 g/dL, Hct: 21%, WBC: 9.2 × 10^9^/lt with 5.7% neutrophil and 70% lymphoid cells, platelet: 64 × 10^9^/lt, ESR: 36 mm/h, ALP: 525 IU/L, GGT: 267 IU/L. Peripheral blood smear showed abnormal lymphoid cells with cytoplasmic projections and these cells were TRAP (+) compatible with HCL.

Bone marrow biopsy showed grade III fibrosis and lymphoid infiltration and these cells were CD20 (+), TRAP (+).

**Management**

Cladribine 0.1 mg/Kg daily for 7 days was given on December 2007. At the last visit on May 2008, his physical examination and complete blood count were within normal limits (Hb: 13.2 g/dL, Hct: 39.4%, WBC: 4.3 × 10^9^/lt, neutrophil count: 2.65 × 10^9^/lt, platelet: 177 × 10^9^/lt.

## 2. Discussion

Urothelial cancer and dye exposure is an old history. The association between chemical exposure and BC is proposed classically but epidemiologic data is variable. When we enter the pubmed with “Bladder cancer and chemical” key words, it shows 795 papers. It has been reported in many papers that every type of exposure to chemicals (from occupational exposure to personal hair dyes or drinking water) may contribute to the occurrence of BC [1–5].

On the other hand, exposure to petroleum products and HCL is another aspect of the chemical exposure and cancer. First report about the chronic benzene exposure and HCL has been presented by Turkish Hematologist Aksoy [6]. After this observation, additional papers have been published about HCL and chemical exposures. However, due to the relative rarity of HCL the data about the association between chemical exposure and HCL, is limited. Male predominance in HCL (4/1) has been noticed by several groups, and this suggested the probability of occupational exposure to ionizing radiation, benzene, and other solvents [7, 8]. In a case-control study covering 291 cases (229 men, 62 women) and 541 controls, there was a positive association between exposures to organic solvents or self-declared exposures to solvents and HCL [9]. In 2 case-control studies only male cases have been included. In the first study covering 111 male cases and 400 controls, there has been found elevated levels of antibodies to the EBV early antigen in HCL cases compared to controls. ORs were found to be elevated in cases with early antigen IgA and to have a history of exposure to environmental solvents, certain pesticides, impregnating agents, animals and exhausts [10]. In the second study, 121 male HCL cases and 484 controls have been included. Elevated OR was found for exposure to farm animals in general (OR: 2.0, CI: 1.2–2.3), exposure to herbicides (OR: 2.9, CI: 1.4–5.9), insecticides (OR: 2, CI: 1.1–3.5), fungicides (OR: 3.8, CI: 1.4–9.9), impregnating agents (OR: 2.4, CI: 1.3–4.6), organic solvents (OR: 1.5, CI: 0.99–2.3), and exhausted fumes (OR: 2.1, CI: 1.3–3.3) showed increased risk. In this study multivariate analysis showed elevated ORs for all these exposures with the exception of insecticides [11]. There are some smaller case-control studies evaluating HCL and chemical exposure. A study covering 45 cases and their neighborhoods evaluated exposure to organic chemicals in the workplace and there was significantly greater exposure in cases than controls (RR: 3.10). Employment in the woodworking or farming was of borderline significance. In this cohort there was a significant risk in farm birthplace (RR: 4.20), migraine (RR: 4.80), infectious mononucleosis (RR: 9), routine use of aspirin (RR: 3.41), and tranquilizers (RR: 4.5) [12]. In another case-control study covering 50 cases and 95 controls, there has been found an important risk between HCL and exposure to organic solvents, petrochemicals, and related products [13]. When evaluated generally, cigarette smoking was associated with reduced or no risk of HCL and this was an interesting point of these studies [8, 11, 12].

In our case there was the history of dye exposure and double cancer that the etio-pathogenetic role of dye has been shown in both. This case is interesting due to 2 reasons:

1. coexistence of HCL and BC in a case with the history of exposure to dye is interesting,
2. epidemiologic data about the dye exposure and HCL is relevant, but we need additional epidemiological studies and/or meta-analysis about chemical exposures and HCL.
